# Research progress of autoimmune diseases based on induced pluripotent stem cells

**DOI:** 10.3389/fimmu.2024.1349138

**Published:** 2024-04-24

**Authors:** Rutong Ren, Jinhe Jiang, Xinxin Li, Guirong Zhang

**Affiliations:** Shandong Yinfeng Academy of Life Science, Jinan, Shandong, China

**Keywords:** autoimmune diseases, multiple sclerosis, inflammatory bowel disease, type 1 diabetes, induced pluripotent stem cells

## Abstract

Autoimmune diseases can damage specific or multiple organs and tissues, influence the quality of life, and even cause disability and death. A ‘disease in a dish’ can be developed based on patients-derived induced pluripotent stem cells (iPSCs) and iPSCs-derived disease-relevant cell types to provide a platform for pathogenesis research, phenotypical assays, cell therapy, and drug discovery. With rapid progress in molecular biology research methods including genome-sequencing technology, epigenetic analysis, ‘-omics’ analysis and organoid technology, large amount of data represents an opportunity to help in gaining an in-depth understanding of pathological mechanisms and developing novel therapeutic strategies for these diseases. This paper aimed to review the iPSCs-based research on phenotype confirmation, mechanism exploration, drug discovery, and cell therapy for autoimmune diseases, especially multiple sclerosis, inflammatory bowel disease, and type 1 diabetes using iPSCs and iPSCs-derived cells.

## 1 Introduction

Autoimmune diseases affect about 8-10% of the world’s population. Autoimmune diseases are caused by the disturbance of immune tolerance to autoantigen, resulting in damage and dysfunction of specific or multiple organs and tissues (1, 2). Organ-specific autoimmune disease, such as type 1 diabetes (T1D), are characterized by lesions and are limited to a specific organ, while systemic autoimmune diseases, such as systemic lupus erythematosus (SLE) and rheumatoid arthritis (RA), involves the pathological damage of multiple organs and tissues due to immune response. Autoimmune diseases decrease the quality of life of patients and social costs. For instance, psoriasis (PsO) is associated with psychological comorbidities, such as depression (3). Inflammatory bowel disease (IBD) patients have a higher level of depression and anxiety than healthy controls (4). Furthermore, autoimmune diseases can cause physical limitations or disabilities in patients with refractory to conventional treatments. About 15% of multiple sclerosis (MS) patients developing into the next stage of secondary progressive multiple sclerosis (SPMS) experience the loss of self-care ability due to the progressive and irreversible accumulation of neurological disability (5, 6). Moreover, RA leads to some gradual symptoms if not timely treated, such as morning stiffness and inflexibility of joint movement, which may lead to permanent disability (7). Although the pathogenesis of autoimmune diseases is unclear, genetic and environmental factors trigger immune dysregulation. Specifically, the major histocompatibility complex (MHC), particularly in MS and T1D, is the strongest genetic risk component of immune dysregulation (8, 9). Non-HLA risk genes such as tumor necrosis factor (TNF) receptor superfamily member 1A (10), interleukin (IL)-2 receptor subunit α (IL-2RA) (11), SH2B adaptor protein 3 (12), and Cytotoxic T-lymphocyte Associated Antigen 4 (CTLA4) (13), are also involved in autoimmune diseases. Among environmental factors, early life exposures, lifestyle and hygiene, infection, vaccinations, surgeries and drug exposure and air pollution are significantly related to the development of autoimmune diseases (1). Immunosuppressive agents, including synthetic and biologic drugs, are widely used for the management of autoimmune diseases. These agents target non-receptor tyrosine kinases [janus kinases (JAKs)], pro-inflammatory cytokines TNF-α and IL-17a, and cell adhesion molecules (integrin a4b1 and integrin a4b7) (1).

Nowadays, as a breakthrough discovery of regenerative medicine in 2006, induced pluripotent stem cells (iPSCs) technology has become a fundamental technology applied in a wide range of fields, including mechanism exploration, phenotype identification, disease model, drug discovery and cell therapy. iPSCs can be generated from biological samples, such as blood, urine, and skin, through a non-invasive procedure, then differentiated into relevant phenotypic cells, including neuronal cells, hepatocytes, cardiomyocytes, insulin-secreting pancreatic β cells, and immune cells. Therefore, isogenic system of different cell types can be established due to this multidirectional differentiation potential of iPSCs to investigate autoimmune diseases (14). In addition, iPSCs or iPSCs-derived cell disease models have confirmed the treatment effect of some drugs, new compounds, or biological agents. Therefore, iPSCs technology can help in understanding disease mechanisms and the development of novel therapeutic strategies. iPSCs may offer healthy functional cells to replenish lost or injured tissue of patients or induce immune tolerance (15, 16). Cells from autologous iPSCs may reduce the risk of immune rejection and relieve diseases caused by single gene defects through gene-editing technology (17). In preclinical studies, iPSCs-derived neural precursor cells (NPCs), intestinal organoids and islet cells were transplanted into animal models with MS, IBD and T1D and showed some therapeutic effects (18–20). Some investigators also presented a strategy of iPSCs differentiation into regulatory T cells (Tregs) and regulatory dendritic cells against autoimmunity in iPSCs-based immunotherapy (21, 22). iPSCs-based cell drugs may be a safer and more effective alternative approach without raising the ethical concerns previously triggered by embryonic stem cells. This paper provides a review of iPSCs-based research on phenotype confirmation, mechanism exploration, drug discovery, and cell therapy for autoimmune diseases, especially MS, IBD, and T1D.

## 2 MS

MS is a complex neuroinflammatory and neurodegenerative disease of immune-mediated inflammatory demyelinating lesions of the central nervous system (CNS) characterized by visual impairment, limb weakness, and ataxia (23, 24). Peripheral activated T cells can penetrate the blood-brain barrier (BBB) into CNS and trigger various immune responses with the myelin. Local antigen-presenting cells (APCs) can also prime naive T cells to recruit more activated lymphocytes, leading to the formation of MS plaque (23, 25, 26). The clinical process of MS has multiple phases, including ∼85% patients with relapsing-remitting multiple sclerosis (RRMS) and ∼15% patients with the next stage of SPMS (5, 6). Besides, ∼15% of cases are classed as primary progressive (PPMS), in which the disease progression is relentless from the onset (5, 6).

### 2.1 Phenotype confirmation and mechanism exploration

#### 2.1.1 MS-iPSCs-derived NPCs show signs of elevated cellular senescence

MS patients-derived somatic cells can generate iPSCs through cellular reprogramming (27–30). These iPSCs can differentiate into several CNS cell types, including neural stem/progenitor cells, neurons, mature astrocytes, oligodendrocyte progenitor cells (OPCs), and oligodendrocytes (OLs) (31, 32). NPCs are self-renewing multipotent cells that induce anti-inflammatory and neuroprotective effects within a demyelinated lesion by regulating OPCs maturation and myelin regeneration. Unlike healthy controls, NPCs from MS cases have compromised stemness and elevated cellular senescence, preventing the maturation of OPCs into OLs, thus diminishing remyelination potential and leading to inflammation aggravation and axonal death (33, 34). However, it is unknown whether myelination defects associated with senescent NPCs in PPMS is a cause or result of PPMS progression (33).

#### 2.1.2 MS-iPSCs-derived OPCs, OLs, and astrocytes

OLs, astrocytes, and microglia are the three main components of glia (35), which play a key role in myelin-related diseases (36, 37). OLs are the myelinating cells of the CNS that are produced from OPCs and can proliferate and migrate to the lesion (36). Many studies have shown that OPCs and OLs can be normally produced from iPSCs of MS patients like healthy iPSCs (36, 38–42). However, it is controversial whether there are differences in OPCs or OLs derived from iPSCs of healthy controls and MS patients. For instance, Starost et al. reported that the proliferation and migration capacities, differentiation and ensheathment of 3D myelinating fibers, proteome, and stress response stimulated by rotenone and peroxynitrite were not significantly different between RRMS and control iPSCs-derived OLs (40). MS-iPSCs-derived OLs can conserve their properties after engraftment in the developing CNS of immune-deficient mice, such as myelinating potential and specific electrophysiological properties, and functionally interact with axons and glia (41). However, Lopez-Caraballo et al. found that MS-iPSCs-derived OPCs-generated conditioned media (CM) lacked certain proteins involved in cell migration and cell-matrix interaction, which might reduce MS-OPCs migration capacity (36). Exogenous addition of activators, such as laminin, bFGF, and down-regulated NG2/CSPG4, may reverse deficient migration of MS-iPSCs-derived OPCs, suggesting that early OPCs migration capacity may be a potential target for restoring remyelination and neurological function (36). Compared with controls, a transcriptome study showed that PPMS-OLs had several differentially expressed genes involved in cell adhesion, apoptosis, and inflammation (42). Notably, the inflammasome component Nlrp2 is highly up-regulated and may be a potential biomarker for disease phenotyping and progression in the cerebrospinal fluid (42).

Astrocytes are glial cells with several protective functions to the CNS, including neuron trophic support, synapse regulation, and BBB integrity (6). Although astrocytes have been successfully differentiated from iPSCs of MS patients (43–46), it is unclear whether MS-astrocytes and healthy controls-astrocytes are significantly different. Some studies have reported that astrocytes from MS and healthy control iPSCs lines had significant glutamate uptake capacity and similar transcriptome triggered by some pro-inflammatory cytokines (44, 45). However, Ghirotto et al. found that MS-iPSCs-derived astrocytes could impair the uptake and enhance the release of glutamate (47). Notablely, Ghirotto et al. observed higher non-mitochondrial OCR, maximal OCR, and proton leak in MS-astrocytes which was in agree with that the cerebrospinal fluid of MS patients induced increased proton leak in cultured neurons (47, 48). Furthermore, MS-astrocytes showed a repression of amino acid and phospholipid metabolism which has also been shown to drive the pathology in mice model, which was reversed by a widely used immunosuppressive drug fingolimod (47, 49, 50).

#### 2.1.3 The effect of astrocyte on exacerbating neuroinflammation induced by the pro-inflammatory cytokines

Astrocytes are heterogeneous with different expression profiles, morphology, and functional characteristics based on different brain regions in healthy controls and patients (51). iPSCs-derived astrocytes can relay inflammation by secondary producing various cytokines after treatment with the pro-inflammatory cytokines and granulocyte-macrophage colony-stimulating factor (GM-CSF). Particularly, TNF-α can up-regulate the MHC I molecules on the surface of astrocytes, making the astrocytes become the potential targets of CD8+ T cells (44). Notably, TNF-α in combination with IL-1β has a significant synergistic effect (44). Also, CM from iPSCs-derived astrocytes can suppress OPCs differentiation after stimulation with TNF and IL-1α due to the down-regulation of myelin and glial differentiation genes and up-regulation of NF-κB signaling pathway genes (45). MS genetic risk variant rs7665090, associated with NF-κB activation, can enhance astrocyte responses (52). Astrocytes from MS patients with rs7665090 risk variant have a higher level of NF-κB dimers p50 and p65 after treatment with a combination of TNF-α and IL-1β with or without IFN-γ, which up-regulates NF-κB downstream genes, particularly complement component 3, a marker of astrocyte toxic phenotype (43). However, the function of astrocytes in the neuroinflammatory diseases is complex. iPSCs-derived astrocytes induce a protective effect by secreting the growth factor neuregulin-1β and leukemia inhibitory factor (LIF) after treatment with TNF-α alone or co-stimulation with IL-1β (44), which suggested that astrocytes were beneficial to the repair of CNS in a strong pro-inflammatory environment. Moreover, Kerkering et al. reported that healthy control or autologous neurons cultured with iPSCs-derived reactive astrocytes from benign MS patients [an Expanded Disability Status Scale (EDSS) score <3 with full earning ability and a disease duration of over 15 years] exhibit less axonal damage than astrocytes from healthy controls and progressive MS patients [a rapid disability accumulation (EDSS > 6) within 15 years of disease duration] after treatment with TNF-α combined with IL-17A (44). This may be due to neurite-protective supernatants from benign MS cases containing a higher level of LIF, transforming growth factor (TGF)-β, and brain-derived neurotrophic factor (BDNF) that are classical neurotrophic factors induced by sustained activation of the JAK/STAT signaling (46). Although iPSCs-derived astrocytes help us understand the roles of astrocytes, their functional heterogeneity and complex subgroups which might be further identified by high-throughput sequencing techniques, limits our ability to apply astrocytes as a therapeutic target.

### 2.2 Cell therapy

Firstly, transplantation of functional NPCs from iPSCs can promote neural function recovery and attenuate disease pathology in animal models of MS (**Table 1**) (18, 53). Mechanistically, LIF secreted by mouse iPSCs (miPSCs)-derived NPCs can promote endogenous OPCs and mature OLs survival, differentiation, and remyelination, but not through cell replacement (18). A xenotransplantation study showed that human iPSCs (HiPSCs)-derived NPCs could be rapidly rejected after intraspinal transplantation in an MS mouse model with no significant recovery. However, histopathological analysis revealed that transplanted animals have reduced demyelination and focal remyelination (**Table 1**) (54). The possible mechanism is that NPCs can reduce T cells infiltration and increase CD4+FOXP3+ regulatory T cells in the CNS (54). Secondly, OPCs and OLs are involved in myelination, indicating that transplantation of iPSCs-derived OPCs and OLs can treat MS in the animal models, whether the donor is healthy or not (**Table 1**) (38, 41, 55, 56). Wang et al. reported that HiPSCs-derived OPCs could proliferate, migrate, and differentiate into mature OLs and produce myelin in the brain of immunodeficient shiverer mice (55). Besides, the speed and efficiency of myelination are higher in HiPSCs-derived OPCs than in fetal tissue-derived OPCs (55). Implanted HiPSCs-derived OPCs can migrate to the lesions and remyelinate denuded axons and survive for at least 40 days in a progressive MS monkey model after treatment of immunosuppressant cyclosporine A (56). Moreover, it is also demonstrated that MS-iPSCs-derived OPCs are functional and produce compact myelin when transplanted into the forebrain of shiverer mice, which give people confidence and hope toward the development of autologous cell therapies using iPSCs (38, 41). But we still consider whether transplanted cells will preserve susceptibility to triggering factors when specific defective mutations are not corrected.

**Table 1:** The therapeutic effects of cell therapy based on iPSCs in the treatment of MS.

Report	Cell used	Transplantation routes	Recipient animal	Key findings
Laterza et al. (18)	miPSCs-NPCs	Stereotaxic injection into cisterna magna	C57BL/6 mice	miPSCs-NPCs exerted the neuroprotective effect via the secretion of LIF leading to a secondary anti-inflammatory role, not due to a cell replacement mechanism in the experimental autoimmune encephalomyelitis (EAE) model.
Zhang et al. (53)	miPSCs-NPCs	Intraventricular injection	C57BL/6 mice	miPSCs-NPCs dramatically reduced T cells infiltration and ameliorated white matter damage in the EAE model.
Plaisted et al. (54)	HiPSCs-NPCs	Intraspinal injection	C57BL/6 mice	The short-lived transplantation of NPCs could dampen neuroinflammation and remyelination with the decrease of CD4+ T cells and the increase of CD4+FOXP3+ Tregs in the MS animal model but not lead to significant clinical improvement.
Douvaras et al. (38)	MS-iPSCs-OPCs	Stereotaxic injection into corpus callosum	Immunocompromised shiverer/rag2 mice	MS-iPSCs-OPCs could differentiate into OLs and form compact myelin *in vivo*.
Mozafari et al. (41)	MS-iPSCs-OLs	Stereotaxic injection into corpus callosum	Immunocompromised shiverer/rag2 mice	MS-iPSCs-OLs could promote remyelination, rescue axon conduction velocity, and functionally connect to other glial cells *in vivo.*
Wang et al. (55)	HiPSCs-OPCs	Stereotaxic injection into corpus callosum	Immunocompromised shiverer/rag2 mice	HiPSCs-OPCs could efficiently differentiate into both myelinogenic OLs and astrocytes *in vitro* and *in vivo*. The myelination speed and efficiency of HiPSCs-OPCs was higher than fetal tissue-derived OPCs.
Thiruvalluvan et al. (56)	HiPSCs-OPCs	Stereotaxic injection into corpus callosum	Common Marmoset	HiPSCs-OPCs could selectively migrate to MS-like lesions and initiate remyelination of denuded axons within the inflammatory conditions of these lesions in the EAE monkey model.

**Figure 1:**
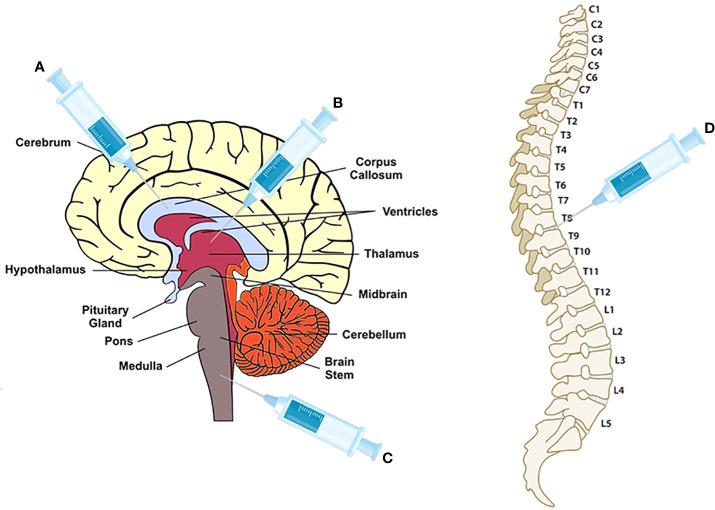
The injection sites for NPCs, OPCs and OLs transplantation. **(A)** corpus callosum for OPCs and OLs transplantation (38, 41, 55, 56); **(B)** ventricle for NPCs (53); **(C)** cisterna magna for NPCs (18); **(D)** vertebra T8-T9 for NPCs (54).

As shown in **Figure 1**, the transplanted sites are cisterna magna (18), ventricle (53), and corpus callosum (38, 41, 55, 56), which need an invasive surgical transplantation except intracranial injection in thoracic T8-T9 (54). Compared to the intrathecal transplantation in a recent clinical study of neural stem cells transplantation in progressive MS patients (57), intracranial injection may have a worse safety and tolerability for patients. In addition, lesion locations of MS are diffusive, including optic nerve, cerebral white matter, corpus callosum, brainstem, cerebellum, and spinal cord white matter (58), it may be impossible to improve all clinical symptoms of MS through a specific area for transplantation. Some research objects, for example, selection of transplant site based on the correlation between lesion location and disability as well as comparison of therapeutic effects among different transplant sites, should be carried out.

### 2.3 Drug discovery

The therapeutic effects of marketed drugs have been verified in iPSCs-based *in vitro* disease model (**Table 2**). Nicaise et al. showed that MS-iPSCs-derived NPCs could secrete high-mobility group box-1, a directly acting inhibitor of OPCs differentiation, thus reducing the regenerative capacity of OPCs within the lesion environment (59). Furthermore, mammalian target of rapamycin (mTOR) inhibitor rapamycin can restore MS-NPCs-mediated support for OLs maturation (**Table 2**) (59). Chronic IFN-γ exposure significantly inhibits differentiation of iPSCs-derived OLs, particularly if the exposure is initiated during the pre-progenitor stage (39). Supernatants of activated PBMCs induced by phytohaemagglutinin (PHA) can impair iPSCs-derived OLs differentiation because of IFN-γ secreted by PBMCs (40). And immunomodulatory drug teriflunomide can partly restore the impairment by reducing the proliferation of immune cells in PBMCs, especially T cells (**Table 2**) (40).

**Table 2:** The therapeutic effects of some chemical or biological molecules were confirmed for autoimmune diseases in the iPSCs-based studies.

Disease	Target	Compound	Drug Approvals or Not in FDA
MS	mTOR	Rapamycin (59)	Approved
	DHODH	Teriflunomide (40)	Approved
IBD	CXCL8/CXCR1 axis	Repertaxin (8)	Phase 3 Clinical
	PTGER2	PF-04418948 (60)	Phase 1 Clinical
	PTGER4	L-161,982 (60)	Not approved
	TNF-α	Infliximab (61)	Approved
	TGF-β	SB431542 (61)	Not approved
	JAK1	Filgotinib (62)	Approved
	MAPK	SB202190 (62)	Not approved
T1D	RNLS	Pargyline (63)	Discontinued
	JAK1/2	Ruxolitinib (64)	Approved
RA	NMNAT3	Tannic acid (65)	Not approved

MS, multiple sclerosis; mTOR, mammalian target of rapamycin; DHODH, dihydroorotate dehydrogenase; IBD, inflammatory bowel disease; CXCL8/CXCR1, the chemokine (C-X-C motif) ligand (CXCL) 8/C-X-C motif chemokine receptor 1; PTGER, prostaglandin E receptor; TNF-α, tumor necrosis factor α; TGF-β, transforming growth factor-β; JAK, janus kinases; MAPK, mitogen-activated protein kinases; RNLS, renalase; T1D, type 1 diabetes; NMNAT3, nicotinamide nucleotide adenylyltransferase 3.

## 3 IBD

IBD is a chronic non-specific intestinal inflammatory disease of the digestive tract and may be caused by an aberrant immune response against the gut microbiota triggered by environmental factors in a genetically susceptible host (66). It is mainly characterized by diarrhea, abdominal pain, and rectal bleeding and about 50% of IBD patients experience extraintestinal manifestations, such as peripheral arthritis, nephrolithiasis, and erythema nodosum (66, 67). IBD is divided into ulcerative colitis (UC) and Crohn’s disease (CD). UC inflammation is limited between colonic mucosal and submucous layer, while CD inflammation may occur in any part of the gastrointestinal tract from mouth to anus in a non-continuous type (66).

### 3.1 Phenotype confirmation and mechanism exploration

#### 3.1.1 IBD patients-derived organoids

Studies have shown that tissue samples from IBD patients can produce iPSCs (68–70), which can differentiate into human intestinal organoids (HIOs) and human colon organoids (HCOs). HIOs and HCOs represent more complex structures and epithelial response characteristics with the complex multicellular environment of the intestinal and colonic tissue to detect disease phenotypes. Hohwieler et al. found that steroid refractory CD patients and a healthy donor-derived HIOs were not significantly different, but detected a slight reduction of the number of goblet cells (71). However, Sarvestani et al. assessed the clinical phenotype of active UC via UC-iPSCs-derived HCOs (8). Compared with healthy controls, they showed that HCOs from UC patients had a significantly higher ratio of non-columnar epithelium, an aberrant rate of epithelial proliferation, significantly reduced acid mucin secretion, and a lower expression of β-catenin and E-cadherin. These results were further verified when the organoids were engrafted into the momentum of immunodeficient mice (8).

#### 3.1.2 The pro-inflammatory cytokines are the core of IBD disease progression

Pro-inflammatory cytokines enhance permeability and formation of fibrosis in the HIOs (72). Disruption of the intestinal epithelial barrier is a hallmark of mucosal inflammation, which could be induced by some inflammatory mediators in HIOs (73). TNF-α and IFN-γ enhance epithelium permeability and changes in tight and adherens junction architecture due to the mis-localization and decreased expression of ZO1 and E-cadherin, instead of inducing cytotoxicity (72, 74). However, only IFN-γ-treated HIOs do not affect the expression or localization of ZO-1 and E-cadherin (75). Transcriptional profiling of HIOs has indicated that many genes, such as indoleamine 2,3-dioxygenase 1 (IDO1) and guanylate binding protein 1 (GBP1), are up-regulated after IFN-γ stimulation, consistent with the results of biopsy tissue from IBD patients (75). Furthermore, Estrada et al. established fibrosis model of mesenchymal stem cells (MSCs) and epithelial cells from iPSCs-derived HIOs treated with the profibrogenic cytokine TGF-β to study intestinal fibrosis, a serious complication of CD. They confirmed that profibrotic genes and mesenchymal-associated genes are significantly up-regulated in MSCs and epithelial cells (76). Interestingly, in both of Gleeson et al. ‘s and Estrada et al. ‘s studies, compared to healthy individuals, CD patients-derived HIOs did not demonstrate a worse outcome on the permeability and profibrotic genes expression respectively induced by a co-stimulation of TNF-α and IFN-γ, or TGF-β alone, which might be explained as low number of subjects coupled with the enormous heterogeneity observed in CD patients (74, 76).

#### 3.1.3 The loss-of-function of anti-inflammatory cytokines is an important driving factor of IBD progression

**Figure 2:**
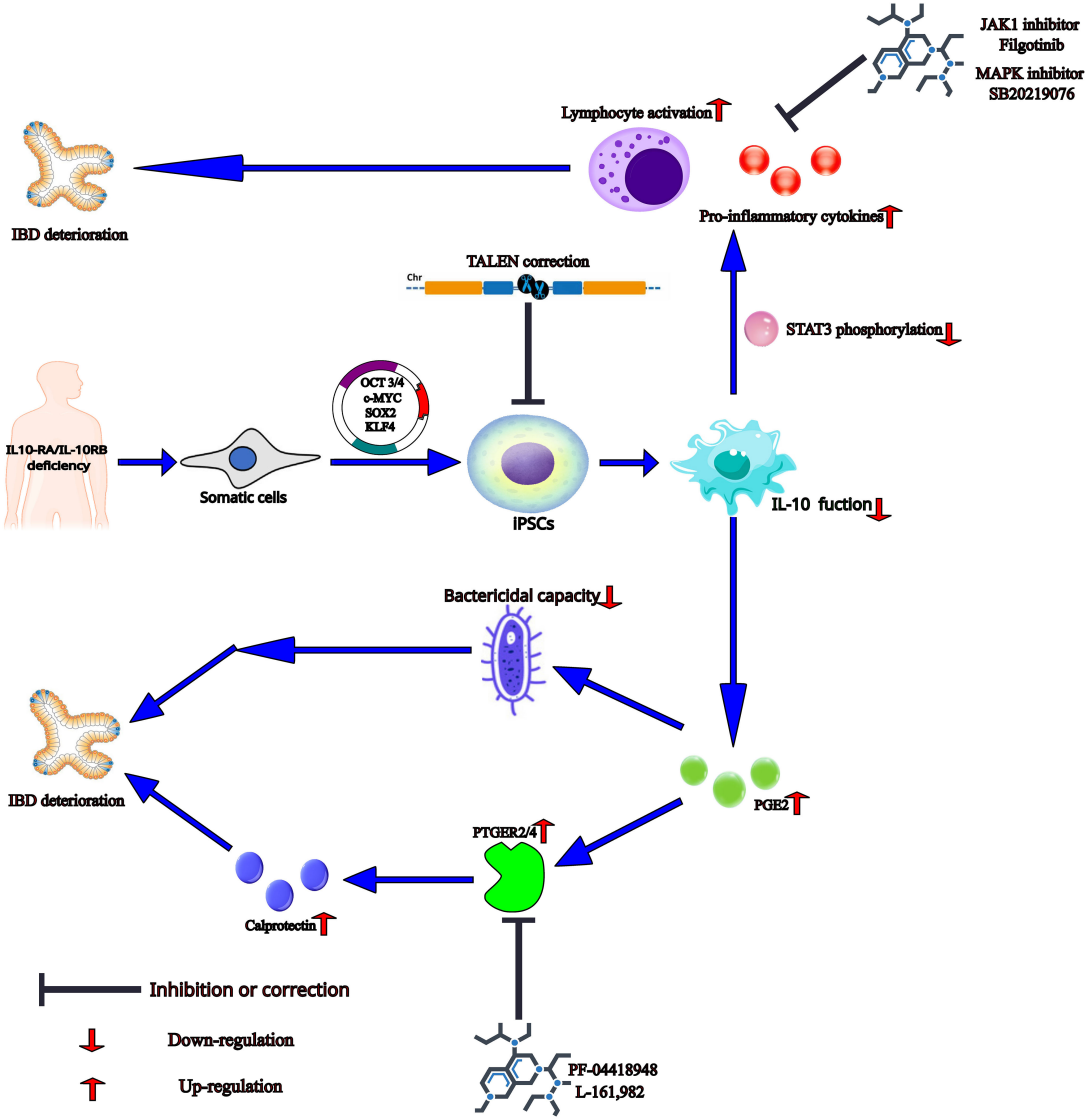
The mechanism and potential treatment methods in the VEO-IBD patients with IL10-RA/IL-10RB deficiency in the iPSCs-based studies. The somatic cells from the patient with IL-10RA/IL-10RB deficiency are reprogrammed to iPSCs by transfected SOX2, KLF4, c-MYC, and OCT3/4. And then patient-derived iPSCs differentiate into IL-10RB-/- and IL-10RA-/- macrophages, which loss IL-10-mediated anti-inflammatory effects, leading to the decrease of STAT3 phosphorylation and the increase of PGE2 production. Correction of iPSCs by gene editing technology such as TALEN will produce normal macrophages with anti-inflammatory function. The dysregulated STAT3 phosphorylation can cause the higher levels of pro-inflammatory cytokines and lymphocyte activation and thereby induce IBD deterioration, which can be inhibited by JAK1 inhibitor Filgotinib and MAPK inhibitor SB20219076. PGE2 may disrupt bactericidal capacity of the defective macrophages, possibly due to a decreased intracellular killing and not decreased phagocytosis ability. In addition, by activating prostaglandin E receptor (PTGER), PGE2 can promote the secretion of calprotectin, which may exacerbate disease and indicate IBD severity. In this stage, blockade of PTGER2 and PTGER4 respectively by PF-04418948 and L-161,982 can rescue the phenotype in IL-10 mutant macrophages.

IL-10 is a key anti-inflammatory cytokine with pleiotropic immunosuppressive functions and mainly targets APCs (77, 78). IL-10 can inhibit the release of pro-inflammatory cytokines by mediating phosphorylation of signal transducer and activator of transcription 3 (STAT3) and its downstream gene BCL3 and SOCS3 expression after binding with its receptors containing two α subunits (IL-10RA and IL-10RB) (60, 62, 79). Furthermore, IL-10RA/IL-10RB deficiency can result in infantile-onset IBD or very early-onset IBD (VEO-IBD) with severe phenotypes due to disruption of IL-10-mediated anti-inflammatory effects, leading to activation of pathogenic macrophages and intestinal inflammation (60, 62, 70, 79). Notably, macrophages can be generated from iPSCs of infantile-onset IBD patients carrying IL-10RA/IL-10RB deficiency, suggesting that IL-10 signaling does not impact the differentiation of iPSCs to macrophage (**Figure 2**) (60, 62, 70, 79). The infantile-onset IBD patient iPSCs-derived IL-10RB-/- macrophages have almost the same gene expression profiles as wild type (WT) macrophages except for ∼25 genes, including SOCS3 and MARCH1, with known anti-inflammatory functions. This finding suggests that IL-10 cannot induce a strong transcriptional response under no stress, even in WT macrophages (60). But lipopolysaccharide (LPS)-induced cytokine secretion such as TNF-α and IL-6 cannot be suppressed effectively by IL-10 without STAT3 phosphorylation and subsequent SOCS3 expression in iPSCs-derived 10RB-/- and IL-10RA-/- macrophages (**Figure 2**). TALEN-mediated genetic correction of VEO-IBD-iPSCs restores anti-inflammatory function in iPSCs-derived macrophages. Furthermore, IL-10RB-/- and IL-10RA-/- significantly impair the bactericidal capacity of infantile-onset IBD iPSCs-derived macrophages, possibly due to a decreased intracellular killing and not decreased phagocytosis ability (**Figure 2**) (60, 62). This aberrant antimicrobial response could be due to the up-regulation of the inflammatory mediator prostaglandin E2 (PGE2), thus promoting IBD progression in children and adult patients (**Figure 2**) (80–83). Given that IL-10 and PGE2 have a negative feedback loop, the dysfunction of IL-10 can promote PGE2 synthesis in IL-10RB-/- and IL-10RA-/- macrophages (60). Also, PGE2 can promote the expression of calprotectin by activating prostaglandin E receptor (PTGER), followed by signal transduction via adenylate cyclase (**Figure 2**) (83). CP is a pro-inflammatory protein whose level in fecal and serum samples can indicate IBD severity (84).

IL-37 is another key suppressor of innate immunity (85). The receptors of IL-37, IL-1R8, and IL-18R1, are highly expressed in the gastrointestinal tract, indicating that IL-37 plays an important role in immune homeostasis of gut (85). IL-37 up-regulation can reduce inflammation and alleviate IBD symptoms in dextran sodium sulfate (DSS)-induced colitis model, indicating that IL-37 or its regulatory factors are a promising target for IBD treatment (86, 87). Zhang et al. found infantile IBD in a 4-month-old boy with a homozygous IL-37 mutation and without other rare variants in known VEO-IBD disease-causing genes (88). Compared with familial healthy control, the iPSCs-derived macrophages of IL-37 mutation patient had a higher level of intracellular IL-37 and increased TNF-α after LPS or IL-1β stimulation, consistent with PBMCs results (88). Further studies demonstrated that mutant IL-37 was easily degraded by endoplasmic reticulum pathway or not properly secreted, indicating that mutant IL-37 cannot reduce the production of the pro-inflammatory cytokines (88). Another study showed that blockade of IL-10 receptor could not affect IL-37-mediated protection (86), indicating that IL-10 and IL-37 exert anti-inflammatory effects independently.

### 3.2 Cell therapy

#### 3.2.1 Effects of HIOs transplantation in an IBD animal model

The conventional pharmacologic agents, such as aminosalicylic acids, glucocorticoids, immunosuppressive drugs, and biological agents, can only control IBD symptoms and cannot completely cure IBD (89, 90). An innovative protocol has been published for the transplantation of iPSCs-derived HIOs into a mouse model of colitis. In this study, two types of HIOs, HIOs-A and HIOs-B, were generated via suspension culture with and without small molecules, including a TGF-β pathway inhibitor (A-83-01), a MEK inhibitor (PD98059), an DNA methylation inhibitor (5-Aza) and a Notch activation blocker (DAPT) (**Table 3**) (20). HIOs-A without coculture with the small molecules had more intestinal stem cells expressing Lgr5, while HIOs-B cultured with the small molecules had more intestinal mature cells, including epithelial and secretory cells (20). Animals in the HIO-A group had a faster recovery (body weight and clinical symptoms with lower acid mucopolysaccharide level and less colonic stricture) than both HIO-B and natural healing group after transplantation into colon tissue of acute colitis mice model induced by DSS (20). In addition, HIO-A and HIO-B decreased the production of pro-inflammatory cytokines in the colon at different degrees (20). Therefore, the strong proliferation and differentiation ability of stem cells is crucial for the repair and regeneration of diseased tissues in cell replacement therapy. However, the cell replacement therapy of intestinal organoids may not be suitable for CD, because CD can affect any part of the gastrointestinal tract. Like MS, it is crucial to select the optimum transplanted area (66). The injection into the colonic lumen is difficult to operate for gastroenterologist and hard to be accepted by patients.

**Table 3:** The therapeutic effects of cell therapy based on iPSCs in the treatment of IBD.

Report	Cell used	Transplantation routes	Recipient animal	Key findings
Nakanishi et al. (20)	HiPSCs-HIOs	Injection into the colonic lumen	NSG mice	HIOs with more intestinal stem cells could promote the alleviation of clinical symptoms and suppress the inflammatory response in the colitis mice model compared with the organoids with more mature intestinal cells.
Soontararak et al. (91)	miPSCs-MSCs	Tail vein injection	CD‐1 mice	miPSCs-MSCs had the equivalent effect with adipose-derived MSCs and normalize the intestinal microbiome in the DSS-induced IBD model.
Kagia et al. (92)	HiPSCs-MSCs	Intraperitoneal injection	C57BL/6 mice	Therapeutic effects of HiPSCs-MSCs were inferior to that of bone marrow/umbilical cord blood-derived MSCs in the DSS-induced IBD model.
Yang et al. (93)	HiPSCs-MSCs	Intraperitoneal injection	BALB/c or C57BL/6J mice	HiPSCs-MSCs could promote epithelial cell proliferation for mucosal healing in a murine colitis model via the interactions of TSG-6 and hyaluronan-CD44 in an Akt-dependent manner.

#### 3.2.2 Reliability of iPSCs-derived MSCs compared with bone marrow and adipose tissue-derived MSCs

Although some studies have published the clinical data of autologous or allogeneic MSCs transplantation in CD or UC based on homing and immunoregulation of MSCs, the rate of clinical remission is uncertain with unclear definitions (90, 94, 95). MSCs can be originated from bone marrow, adipose tissue, umbilical cord, and iPSCs. iPSCs-derived MSCs have almost identical phenotypes and tri-lineage differentiation ability with adipose tissue and bone marrow-derived MSCs (**Table 3**) (91, 92) and share approximately 90% of their transcriptome with primary MSCs derived from bone marrow (96). However, the preclinical studies on the therapeutic effects of iPSCs-derived MSCs have shown conflicting results in the animal model of IBD. Soontararak et al. found that iPSCs-derived MSCs (via tail vein injection) and adipose tissue-derived MSCs could significantly improve clinical abnormalities, reduce lesion scores and inflammation in the gut of DSS-induced mice colitis model (91). iPSCs-derived MSCs can promote epithelial regeneration with increasing Lgr5+ intestinal stem cells and reverse microbiome dysbiosis but cannot affect lymphocytic infiltrate in colonic tissues (91). It was surprising that few MSCs mainly located in colonic tissues, suggesting that infusive MSCs might not directly act on colonic epithelial cells, but depend on paracrine secreted pathway for therapeutic effects (91). Yang et al. also observed the iPSCs-derived MSCs promoted mucosal healing by increasing the proliferation of intestinal epithelial stem cells (Lgr5+ and CD44+ cells) in the trinitrobenzene sulfonate (TNBS) and DSS-induced mice colitis model via secreting TNF-α-stimulated gene-6 (TSG-6), which has been proved to have potential immunoregulatory capacity and tissue protective effects in the disease models such as myocardial infarction, arthritis, atherosclerosis, wound healing and fibrosis (**Table 3**) (93, 97). In the study, the coculture system consisting of ex vivo murine/IBD patient-derived HCOs and iPSCs-derived MSCs showed that iPSCs-MSCs could promote the proliferation of colonic epithelial cells and increase cycling crypt base columnar stem cells through TSG-6-mediated hyaluronan-CD44 interactions in an Akt-dependent manner. In contrast, CD44 inhibitory peptide (PEP-1) or TSG-6 knockdown in iPSCs-MSCs can abrogate the above effect (93). However, another study revealed that MSCs derived from iPSCs (via intraperitoneal administration) can significantly prolong the survival of mice with DSS-induced acute IBD, but not significantly reverse clinical symptoms and histopathological changes compared with bone marrow and umbilical cord-derived MSCs (**Table 3**) (92). This could be because iPSCs-derived MSCs have more immature subpopulations of cells compared with tissue-derived MSCs, which experience a pluripotent state (92). In fact, we should consider aging or late passages could reduce proliferative and migration capacity, differentiation potential, and anti-inflammatory function (96, 98), but the authors did not report the iPSCs-derived MSCs passages in the experiment (92).

### 3.3 Drug discovery

In this section, the effects of some chemical and biological targeted drugs on IBD are evaluated in the ‘disease in a dish’ based on iPSCs. Onozato et al. developed a TNF-α and TGF-β-induced HIOs model of IBD with up-regulated mesenchymal and fibrosis markers (61). They observed that anti-TNF-α antibody infliximab and the TGF-β signaling pathway inhibitor SB431542 have therapeutic effects, suggesting that iPSC-derived HIOs can be used as a model of epithelial damage and fibrosis for drug screening (**Table 2**) (61). Sarvestani et al. confirmed that the function abnormality of the chemokine (C-X-C motif) ligand (CXCL) 8/C-X-C motif chemokine receptor (CXCR) 1 axis could promote the progression of UC phenotypes during organoid development, which could be relieved by the small-molecule non-competitive inhibitor repertaxin (**Table 2**) (8). For functional deficiencies of IL-10, on the one hand, an approved JAK1 inhibitor Filgotinib and unapproved mitogen-activated protein kinases (MAPK) inhibitor SB20219076 can reduce the secretion of pro-inflammatory cytokines in IL-10R knock-out iPSCs-derived macrophages (**Figure 2**, **Table 2**) (62). On the other hand, pharmacological blockade of PGE2 synthesis and its receptors PTGER2 (PF-04418948) and PTGER4 (L-161,982) can rescue the phenotype of bacterial killing caused by increased PGE2 in IL-10 mutant macrophages (**Figure 2**, **Table 2**) (60).

## 4 T1D

T1D is a chronic autoimmune disease requiring long-term dependence on exogenous insulin treatment due to immune-mediated destruction of pancreatic β cells. T1D may also lead to other complications, such as diabetic nephropathy, diabetic neuropathy, and retinal dystrophy (99).

### 4.1 Phenotype confirmation and mechanism exploration

#### 4.1.1 T1D-iPSCs-derived β cells/insulin-producing cells/pancreatic progenitor cells can produce insulin in response to glucose

iPSCs can be generated from somatic cells of T1D patients (100, 101), and further differentiate into pancreatic β cells (19, 102), cardiomyocytes (103), and macrophages (104). Studies have shown that T1D-iPSCs-derived β cells/insulin-producing cells/pancreatic progenitor cells express β cell markers (NKX6-1 and Pdx1) and have global gene expression patterns like β cells from control iPSCs (19, 105, 106). However, results showed that pancreatic lineage-specifying and pluripotency-associated factors had intrapatient variations of the expression level and time in the differentiation stage from iPSCs to β cells between T1D patients and normal controls (105). Also, these cells can produce mature endocrine secretory granules (107), increase insulin release in response to glucose (102, 108), and control glucose level in diabetic mice after transplantation *in vivo (* 19). One article in Pubmed reported that iPSCs-derived from T1D patients had a lower efficiency in functional pancreatic β cells and Pdx1 expression compared with nondiabetic iPSCs (109). Moreover, treatment with a potent demethylating agent demethylation 5-Aza-DC can relieve this phenomenon. Besides, these cells can produce insulin-like cadaveric β cells and relieve hyperlipemia in diabetic mice after successful transplantation (109). A recent study reported that iPSCs-derived β cells from healthy volunteers had a slightly higher average value of insulin after glucose stimulation than T1D-β cells (14). In summary, most studies have demonstrated that insulin secretion ability and β cell marker expression are not significantly different between insulin-producing cells derived from T1D and normal control. But under stressed condition, T1D-iPSCs-derived insulin-producing cells show the vulnerability to the inflammatory cytokines insults (110). Hosokawa et al. found that fulminant T1D (a subtype of T1D characterized by a drastic onset of hyperglycemia and all pancreatic β cell destruction)-iPSCs-derived insulin-producing cells were susceptible and have the overexpression of apoptosis associated genes to co-stimulation of TNF-α, IL-1β, and IFN-γ. And fulminant T1D-iPSCs-derived insulin-producing cells have a higher ratio of apoptotic cells than healthy iPSCs-derived cells (110). RNA sequencing and gene set enrichment analysis have been used to detect the differential expression level of interferon-stimulated gene CH25H and B cells differentiation-related genes between fulminant T1D and control samples (110). This indicates that fulminant T1D-derived β cells may not have the antiviral function and immunoregulation function, thus inducing rapid β cell destruction and disease development (110).

#### 4.1.2 β cells are attacked by the immune system in an antigen-dependent manner based on cellular interactions model of iPSCs-derived immune cells

T1D is caused by the breakdown of immune regulation, resulting in infiltration of innate immune cells, migration of autoreactive T cells, and production of autoantibody by B cells, thus destroying the insulin-producing β cells (111). Armitage et al. derived an isogenic system consisting of T cells, endothelial cells, macrophages, dendritic cells, and β cells from a single donor iPSCs line (14). T1D pathogenic cell-cell interactions in this system, including T cells activation and expansion, trafficking into pancreatic islets across endothelial cells (EC) lining capillary walls, and targeted destruction of β cells by lymphocytotoxicity, depend on an antigen-specific manner (14). Joshi et al. confirmed that T1D-iPSCs-derived macrophages could present islet-specific antigen, including proinsulin C-peptide, peptide 11, and islet extract to islet-infiltrating CD4^+^ T cells from the same donor along with CD69 (T cells activation marker) up-regulation (104). HLA-DQ blocking antibody significantly reduces CD69 up-regulation, while HLA-DR blocking antibody does not affect CD69 (104). Moreover, a similar study showed that iPSCs-derived β cells could not activate T cells in the condition of endoplasmic reticulum stress induced by thapsigagin when HLA class I was blocked and knocked out, indicating that HLA molecules play a key role in the destruction of pancreatic β cells and result in sustained inflammation in islet (112). This may explain why HLA is the most susceptible risk gene in T1D (112). An unexpected result showed that iPSCs-derived β cells were prone to be selectively killed by T cells compared to cardiomyocytes and iPSCs-derived α cells, because β cells are highly secretory cells and more susceptible to endoplasmic reticulum stress (112). Using the multi-directional differentiation potential of iPSCs, these studies successfully established a model of cell interaction to imitate the process of β cell destruction in T1D pathogenesis, which is a valuable tool, being highly recommended in the research on other autoimmune diseases.

#### 4.1.3 T1D-iPSCs differentiation into functional cardiomyocytes

Numerous studies have pointed out that individuals with diabetes face a 2 to 4 times higher risk of cardiovascular disease compared to healthy individuals (113). Kikuchi et al. found that T1D-iPSCs exhibited a comparable proliferation capacity and could differentiate into spontaneously contracting cardiomyocytes, displaying three types of cardiac action potentials, along with normal responsiveness to β-adrenergic stimulation (103). Notably, the rate of atrial-like cardiomyocytes was lower compared to cardiomyocytes derived from normal iPSCs (103). Additionally, both normal iPSCs and T1D-iPSCs differentiated cardiomyocytes demonstrated well-regulated glucose metabolism, including glycolysis, and the ability to switch metabolic pathways independently of extracellular glucose concentration (103).

### 4.2 Cell therapy

#### 4.2.1 Addressing the critical issues in the transplantation of iPSCs-derivatives

**Figure 3:**
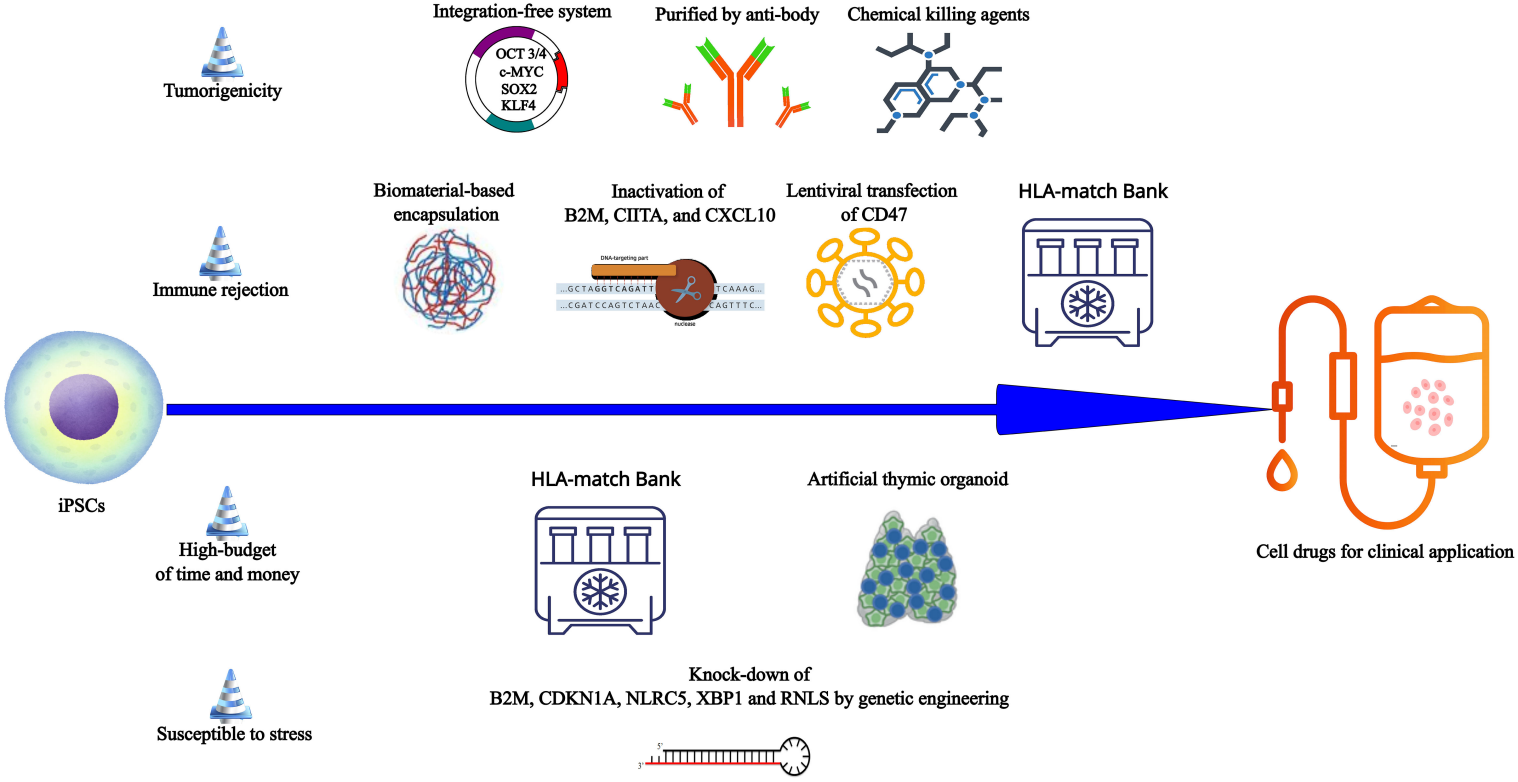
The obstacles and potential corresponding solutions in the way from iPSCs to cell drugs for clinical application. From iPSCs to the qualified cell drugs, four challenges must be addressed. **(i)** The integration-free reprogramming system (Sendai viral delivery, plasmid transfection and chemical reprogramming) can reduce the risk of tumorigenicity because these systems cannot induce genomic instability or insertional mutagenesis. Target cells by purified by anti-body and undifferentiated iPSCs eliminated by chemical agents may ensure the purity of target cells and reduce tumorigenesis. **(ii)** Encapsulation based on biomaterials may prevent iPSCs-derivatives from the destroy of various immune cells to suppress the immune rejection. Manipulation of Beta-2-Microglobulin (B2M), MHC class II transactivator (CIITA), the chemokine (C-X-C motif) ligand (CXCL) 10 (CXCL10), and CD47 can make iPSCs derivatives escape immune clearance. The bank which has thousands of discrete HLA-typed iPSC lines can provide adequate matching for most individuals. Allogeneic HLA-matched cell transplantation may reduce the allogeneic graft rejection. **(iii)** To save the high-budget of time and money in the autologous personalized transplantation therapy, patients can obtain the HLA-matched iPSCs from the bank to produce the desired cells whenever necessary. In the immunotherapy based on iPSCs, artificial thymic organoid provides a large-scale production way to produce the immunocytes such as Tregs. **(iiii)** Inactivation of B2M, cyclin-dependent kinase inhibitor 1A (CDKN1A), NOD-, LRR- and CARD-containing 5 (NLRC5), X-Box Binding Protein 1 (XBP1), and renalase (RNLS), all of which are related with different apoptosis mechanisms, may promote the iPSCs derivatives survival in the host.

Approximately 44% of patients post-islet transplantation can achieve glycemic control independently of exogenous insulin. However, the lack of donors hinders the development of islet transplantation. In an open-label clinical phase 1/2 trial, 17 subjects with T1D received transplants of macro-encapsulated human embryonic stem cells-derived islet cells, with 6 cases showing positive C-peptide levels in serum for 6 months (114). In contrast to embryonic stem cells, iPSCs pose no inherent ethical problems. Pre-clinical studies have demonstrated the functionality of T1D-iPSCs-derived β cells both *in vitro* and *in vivo*, offering opportunities for autologous transplantation (**Table 4**) (19, 109). Nevertheless, challenges persist in HiPSCs derived-islet cells transplantation (**Figure 3**). (i) One primary concern is tumorigenicity from the residual undifferentiated iPSCs and genetic mutations in the reprogramming process (106, 119). (ii) Patient-specific new β cells derived from iPSCs, even when autologous, could face attacks from a recurrence of the autoimmune response due to the expression of neoantigens and/or epigenetic changes (120). (iii) The personalized treatment of autologous iPSC requires a significant economic and time investment (120, 121). (iiii) iPSCs-derived islet cells have the similar susceptibility to stress and inflammatory responses, even more sensitive than islet cells from donor (64, 122).

**Table 4:** The therapeutic effects of cell therapy based on iPSCs in the treatment of T1D.

Report	Cell used	Transplantation routes	Recipient animal	Key findings
Millman et al. (19)	T1D-iPSCs-β cells	Kidney capsule transplantation	Immunodeficient SCID/Beige mice	T1D-iPSCs- and normal iPSCs- derived β cells had the same response to glucose and anti-diabetic drugs both *in vitro* and *in vivo.*
Manzar et al. (109)	T1D-iPSCs-insulin-producing cells	Kidney capsule transplantation	Immunodeficient rag2 mice	T1D-iPSCs displayed a lower differentiation efficiency with poor Pdx1 expression which was restored by a potent demethylating agent. Also, T1D-iPSCs-insulin-producing cells were functional and glucose-responsive *in vitro* and *in vivo*.
Haller et al. (115)	HiPSCs- pancreatic progenitors	Dorsolateral transplantation	Immunodeficient SCID/Beige mice	Macroencapsulated HiPSCs-derived pancreatic endoderm could further differentiate into glucose-responsive islet-like cells and protect mice from STZ-induced hyperglycemia, but this study cannot prove immune rejection effect of microencapsulation.
Montanucci et al. (116)	HiPSCs-β-like cells	Intraperitoneal injection	CD1 mice	After microencapsulation, ELRs coating could protect HiPSCs-β-like cells from immune destruction of host in a 42 days-term post-transplant compared to 48 hours of the uncoated β-like cells.
Hu et al. (117)	HiPSCs/rhesus macaques-islet cells	Dorsolateral transplantation for monkey; Intramuscular injection into the hindlimb muscle for mice	humanized NSG-SGM3 mice and rhesus macaques	Through CRISPR-Cas9 inactivation of B2M and CIITA and lentiviral transfection carrying CD47 gene, the engineered HiPSCs-derived islet cells survived in immunocompetent, allogeneic diabetic humanized mice for 4 weeks and ameliorated diabetes at least 29 days and rhesus macaques hypoimmune iPSCs-derived islets survived for 40 weeks in an allogeneic recipient without immunosuppression.
Haque et al. (118)	miPSCs-Tregs	Tail vein injection	B6-mOVA×OT-I TCR double-Tg mice	Autoantigen-specific iPSCs-Tregs could suppress the migration and activity of the pathogenic immune cells through down-regulating the expression of ICAM-1 in the local inflamed tissues and inhibiting the production of pro-inflammatory cytokine IFN-γ.

#### 4.2.2 A safe reprogramming method is an initial step to mitigate the tumorigenicity risk associated with iPSCs

A previous study demonstrated that the transplantation of pancreatic progenitor cells derived from T1D-iPSCs, reprogrammed via lentiviral vectors, resulted in teratoma formation in over 90% of immunodeficient mice. Remarkably, this issue persisted despite attempts to mitigate it through trypsinization treatment (106). In contrast, pancreatic progenitor cells originating from T1D-iPSCs subjected to nonintegrating Sendai viral reprogramming and enzymatic dissociation exhibited the ability to regenerate into human islet-like structures without teratoma formation at 8 months post-transplantation (106). Consequently, the use of Sendai virus and episomal plasmids emerges as a preferable strategy for limiting tumorigenicity (**Figure 3**). Following the establishment of a safe reprogramming method, it is important to purify target cells or eliminate residual iPSCs. One method involves the use of antibodies that selectively bind to proteins expressed on the surface of pancreatic cells (**Figure 3**) (123–125). However, it is noteworthy that this approach poses a risk of inducing mechanical injury to cells, potentially reducing their viability. An alternative approach involves the application of chemical killing agents to eradicate contaminant iPSCs in the final cell product, using chemical inhibitors (**Figure 3**) (126, 127). These methods are still in the nascent stages of basic research stage and have not yet been incorporated into clinical or preclinical studies focused on iPSCs-derived β cells/pancreatic precursor cells.

#### 4.2.3 The use of a biomaterial-based encapsulation technique holds promise for conferring an immunoprotective role in β cells replacement

This encapsulation technique is employed in islet transplantation to inhibit immune rejection by preventing the infiltration of immune cells and antibodies across a semi-permeable membrane (**Figure 3**). Simultaneously, it allows the free exchange of oxygen, nutrients, and biologically active substances, ensuring optimal conditions for β cell differentiation (128, 129). Haller et al. successfully microencapsulated HiPSCs-derived pancreatic progenitors using hydrophilized PTFE membranes (**Table 4**) (115). Post-implantation into the dorsolateral skin of immunodeficient mice, these pancreatic progenitors underwent a process of differentiation into insulin-positive cells, evidenced by a gradual increase in human C-peptide levels during fasting or glucose stimulation over 18 weeks (115). Notably, in instances where murine β cells were destroyed by streptozotocin (STZ), animals treated with transplantation maintained basal blood glucose levels for several weeks post-STZ (115). One limitation of this study is that HiPSCs-derived pancreatic progenitors are transplanted to the immunecompromised mice, which is not enough to prove immune protection of hydrophilized PTFE (115). It is a problem that pancreatic progenitors from large-scale production had an effective blood glucose control one month later than the small-scale production. The large-scale production tended to influence the maturation efficiency, which seems to be a common problem in extended culture of cell products (115). In a rigorous study, Montanucci et al. encapsulated HiPSCs-derived β-like cells within a microencapsulation system using human elastin-like recombinases (ELR), which did not impede β cell differentiation (**Table 4**) (116). To validate the immune barrier function of ELR, ELR-coated cell spheroids, and uncoated cell spheroids were injected into the abdominal cavity of mice with normal immune function. The results demonstrated that microencapsulated cell spheroids survived with normal morphology and viability, producing insulin without evident signs of inflammation after 42 days. Conversely, within 48 h post-graft, uncoated aggregates were cleared by infiltrating lymphocytes and macrophages (116). Consequently, the authors suggest that ELR appears to provide immunoprotection for the allotransplantation of β cells (116).

#### 4.2.4 Genetic engineering of allogeneic iPSCs prevents immune rejection and apoptosis

One strategy for evading immune rejection is to induce tolerance through genome editing techniques, such as disruption of HLA and overexpression of PDL1-CTLA4Ig fusion (117, 130). In one study, Hu et al. prepared HiPSCs with HLA class I/II molecules deficiency and CD47 overexpression respectively through CRISPR-Cas9 inactivation of Beta-2-Microglobulin (B2M) and MHC class II transactivator (CIITA), and lentiviral transfection of CD47 gene to generate hypoimmune iPSCs (**Table 4**, **Figure 3**) (117). When wild HiPSCs and hypoimmune HiPSCs were subcutaneous injected to the backs of rhesus macaques in a cross-over design, a third injection of the other iPSCs, the engineered HiPSCs did not cause T cells activation, specific antibodies production and NK cells killing, even if the animals received wild HiPSCs injection at the first and second injection (117). And then hypoimmune HiPSCs-derived pancreatic β cells were transplanted into immunocompetent allogeneic humanized mice (117). These transplanted cells could survive at least 29 days and exert the function of glycemic control in response to fasting and glucose (117). Rhesus macaques hypoimmune iPSCs-derived islets survived for 40 weeks in an allogeneic rhesus macaque recipient without immunosuppression (117), indicating this strategy offers a probability for large-scale manufacturing of off-the-shelf cell products without immune rejection. In addition to B2M and CIITA, the knock-out of an IFN-induced chemokine CXCL10 was also proved to prevent stem cell-derived islets from immune cell destruction (131). In fact, there are still other protocols to allograft rejection, including immunosuppressive drugs and MSCs or Tregs immunotherapy (132, 133). Compared with other methods, genetic engineering of allogeneic iPSCs can put things right once and for all, but still need many clinical trials to ensure safety.

To inhibit apoptosis of β cells, Leite et al. reduced the expression of B2M, cyclin-dependent kinase inhibitor 1A (CDKN1A), NOD-, LRR- and CARD-containing 5 (NLRC5), and X-Box Binding Protein 1 (XBP1) individually or together in the iPSCs-derived islet cells through lentiviral small hairpin RNA (shRNA) plasmids (**Figure 3**) (122). Each gene participates in different apoptotic mechanisms, such as endoplasmic reticulum stress and lymphocyte killing (122). The results revealed that knock-down did not damage the function and homeostasis of β cells and individual or all shRNA could protect iPSCs-derived islet from stress-mediated apoptosis and dysfunction induced by cytokine cocktail (IL-1β+TNF-α+IFN-γ), thapsigargin, and high glucose. Encouragingly, individual or all shRNA could suppress the T cell activation and pro-inflammatory cytokines production under the co-culturing stem cells-islets with allogeneic PBMCs (122). Moreover, renalase (RNLS) is a target of iPSCs-derived β cells against endoplasmic reticulum stress and lymphocyte killing (**Figure 3**) (63). In summary, genetic engineering provides a possible solution for survival of transplanted β cells. But we cannot be overly optimistic because there are only animal and *in vitro* experiments in both studies and the safety of several candidate genes knock-down is still unknown.

At last, another strategy to solve the issues of immune rejection is to establish iPSC HLA-match allogeneic cell lines bank for commercialization. The fact is that almost 1500 alleles have been identified at 12 HLA loci and additional alleles are still being discovered, which suggests that this approach requires generating hundreds to potentially thousands of discrete HLA-typed iPSC lines to ensure adequate matching for most individuals (134). Although it requires a significant amount of time and cost to set up a commercialization bank in the GMP system, it will save time and cost compared to autologous personalized therapy (**Figure 3**).

#### 4.2.5 Several potential strategies for enhancing the differentiation efficiency of inducing iPSCs into β cells

The challenge of achieving optimal differentiation efficiency is paramount for researchers aiming to facilitate the transplantation of iPSCs-derived β cells into patients with T1D. There exist several possible approaches to improve this efficiency. Firstly, it has been established that miR-375 and miR-186 act as effective regulators of pancreatic function and the differentiation of islet-like cell clusters (135, 136). Consequently, Shaer et al. and Lahmy et al. attempted to develop a method involving the transfection of miR-186 and miR-375 in the absence of growth factors (136, 137). Remarkably, compared to 30 days required by the conventional four-stage protocol using growth factors, this method facilitated the generation of islet cells within just two days, expressing patterns of pancreatic endocrine-related genes and producing insulin upon glucose challenge. The insulin secretion level by this method was comparatively lower at least 10 times than the conventional protocol (137). Haste makes waste, so this miRNAs transfection way is not advisable.

Extracellular vehicles (EVs) can regulate intercellular communication, signal transmission, and angiogenesis through EV-carried miRNA transfer from donor to recipient cells (138). In a study by Bai et al., mature β cells-derived EVs were introduced into the four stages-protocol of β cells differentiation, resulting in a saving 15 days with high insulin secretion level both *in vitro* and *in vivo* under glucose stimulation (139). Mechanistic studies further revealed that miR-212/132, carried by EVs, functioned as biologically active molecules, reducing the degradation of Ngn3 (an essential transcription factor for β cells derived from stem cells) through binding to the ubiquitin ligase FBW7. Additionally, Ngn3 exhibited a positive feedback loop, promoting the transcription of miR-212/132 by interacting with Pdx1, another critical transcription factor in pancreatic development (139). But the EVs are isolated from human β cells in this study, which are acquired from a pancreas from the deceased donor without diabetes. And EVs contain complex and unclear chemical compositions, which are unfavorable for process stability of β cells differentiation. The chemically defined and controlled conditions are necessary for the iPSCs differentiation and culture to ensure the efficacy and safety in clinical application (140, 141).

Using biocompatible and biodegradable polymer that mimics the microenvironment within the pancreas, the 3D cultivation mode is applied to enhance the differentiation of islet-like cells from iPSCs (142, 143). Both studies identified two advantages including higher levels of pancreas-specific transcription factors and insulin secretion levels in the glucose challenge test (142, 143) in 3D cultivation. Several other strategies are employed to promote the differentiation of iPSCs into β cells. Enderami et al. discovered that treatment with platelet-rich plasma increased the expression of islet genes during the differentiation of iPSCs into insulin-producing cells, reducing the differentiation time from 30 days to 22 days (144). This effect may be attributed to the presence of various growth factors in the cytoplasmic granules of platelet-rich plasma, imparting a high potential for regeneration (145). But this way is not worth spreading and applying because the media containing platelet-rich plasma cannot meet the chemically defined and controlled standard (140, 141).

#### 4.2.6 iPSCs-derived Tregs present a novel avenue for cellular immunotherapy in the treatment of T1D

Tregs, also referred to as suppressor T cells, can inhibit T cell proliferation and cytokines production. In two phase 1 clinical studies, the transfusion of autologous peripheral blood-isolated-polyclonal Tregs demonstrated remarkable survival, persisting over 1 year, and effectively ameliorated symptoms of hyperglycemia (146, 147). Importantly, no significant safety concerns regarding drug-related issues such as broad immunosuppression were identified in patients undergoing Tregs treatment (132). iPSCs can be induced to differentiate towards functional Tregs through the transfection of forkhead box P3 (FoxP3) and antigen-specific T cell receptor (TCR) under stimulation with the Notch ligand (118, 148, 149). Like natural Tregs, iPSCs-derived Tregs can produce immunosuppressive cytokines TGF-β and IL-10 but not IL-2 and IFN-γ in response to anti-CD3/anti-CD28 antibody or specific antigen (22, 118, 148). Haque et al. established a T1D mice model characterized by the destruction of OVA-overexpressing pancreatic β cells due to a substantial influx of activated tissue-associated CD8+ T cells (**Table 4**) (118). Following the adoptive transfer, autoantigen OVA-specific iPSCs-Tregs, but not control cells or non-tissue-associated SM1-specific iPSCs-Tregs, could prevent the migration of pathogenic CD8+ T cells into the pancreas and significantly improve blood glucose levels in all diabetic mice (118). iPSCs are an ideal source of Tregs and genetic engineering of allogeneic iPSCs provides a new approach for producing ‘off-the-shelf’ Tregs, and some studies have reported that 3D-thymus organoid culture based on iPSCs could solve large-scale production of allogeneic Car-T cells to save time and money for patients (**Figure 3**) (150, 151).

### 4.3 Drug discovery

In the study using T1D-iPSCs-derived β cells, Cai et al. confirmed RNLS as a potential target for β cell protection in T1D, aiming to mitigate immune recognition of β cells (63). RNLS, responsible for degrading circulating catecholamines and regulating blood pressure (152), was first discovered as a susceptible factor in autoimmune destruction of pancreatic β cells through a GWAS study of monocytes, including their derivatives, such as macrophages and dendritic cells, from T1D patients (153). Cai et al.’s study demonstrated that RNLS deletion did not affect the ability of iPSCs into β cells and secrete insulin (63). In contrast, RNLS deficiency protected iPSCs-derived β cells against thapsigargin-induced endoplasmic reticulum stress and apoptosis (154). *In vivo* experiments with NOD mice with long-duration diabetes further suggested that treatment with a monoamine oxidase (MAO) inhibitor, pargyline, mimicked the effects of RNLS deficiency, indicating that MAO-inhibitors targeting RNLS could be a promising approach for further research in T1D drug development (**Table 2**) (63).

Pro-inflammatory cytokines secreted by immune cells not only promote T cells activation for β cells destruction but also directly induce apoptosis and dedifferentiation of β cells. Exposure to IFN-γ plus IL-1β or IFN-α induced apoptosis in iPSCs-derived pancreatic endocrine cells, exhibiting a pro-inflammatory and partially dedifferentiated phenotype of β cells, characterized by an increase in CXCL10 and HLA expression and a decrease in the expression of genes encoding pancreatic hormones and key transcription factors crucial for maintaining the β cells phenotype (64). Subsequent studies revealed that Ruxolitinib, a JAK inhibitor primarily used in myelofibrosis treatment, prevented apoptosis and reduced inflammation marker expression induced by IL-1β plus IFN-γ or IFN-α- in islet endocrine cells derived from iPSCs by blocking the STAT1/STAT2 signaling pathway (**Table 2**) (64).

## 5 SLE, RA, Sjogren’s syndrome, ankylosing spondylitis, and PsO

### 5.1 SLE

SLE is a diffuse connective tissue disease mediated by the autoimmune system, characterized by the activation of B cells and T cells and the production of autoantibodies, which may result in damage to the skin, kidney, heart, and CNS (155). Significant efforts have been devoted to elucidating the mechanisms of SLE based on iPSCs derived from SLE patients (156–160). In these studies, no differences in the programming efficiency of somatic cells were observed between SLE patients and healthy controls. However, gene expression analysis showed that SLE-iPSCs had thousands of differentially expressed mRNAs, miRNAs, and proteins in SLE-iPSCs as compared to healthy controls, all consistently related to nuclear constituents and transmembrane transport in biological processes (157). Further research showed that the dysregulation of AK4 protein-miR-371a-5p axis, Erk pathway and Akt pathway might contribute to the progression of SLE pathology (157, 158). SLE-iPSCs exhibited increased sensitivity to oxidative damage, possibly attributed to impaired miR-424 expression, which normally protects neurons by stabilizing the transcription factor erythroid 2-related factor 2 (Nrf2) against oxidative stress (158), which could be a promising therapeutic target of SLE because several Nrf2 signaling activators have been reported to prevent oxidative stress damage (161, 162). Regrettably, subject investigated in both studies was iPSCs, which did not belong to the somatic cells associated with SLE pathology and was not suitable for functional verification (157, 158). Natsumoto et al. further prepared CD123+ dendritic cells from iPSCs derived from SLE patients with 2’-5’-oligoadenylate synthetase like (OASL) variants and established a linkage of functional disturbance and mutant genotype (163). The SLE-dendritic cells with mutant OASL could secrete a higher level of IFN compared to healthy controls in challenge of dsRNA, a biomarker of SLE pathology. In addition, SLE-iPSCs-derived cardiomyocytes exhibited abnormalities, including abnormal sarcomeric structures without filaments, weaker calcium signals, lower proliferation rates, increased hypertrophy, and fibrosis markers protein expression levels induced by the serum derived from SLE patients and anti-Ro autoantibody (164), which suggested that we should pay attention to cardiac and vascular complications leading to death in patients with SLE.

In the cell therapy, allogeneic cord blood-derived Tregs and umbilical cord tissues/placenta-derived MSCs can improve SLE symptoms in preclinical and clinical studies (165, 166), but there have not been the related studies on treatment effect of SLE by Tregs or MSCs derived from iPSCs.

### 5.2 RA

RA is a chronic inflammatory condition, primarily characterized by joint involvement, systemic inflammation, and several comorbidities (7). Fibroblast-like synoviocytes (FLSs) are often obtained for iPSCs reprogramming during arthroscopic synovectomy or total knee replacement surgery (65, 167–171). In these studies, RA-iPSCs-derived somatic cells always displayed some abnormal properties in varying degrees compared to healthy controls, including higher proliferation rate and greater energy consumption in FLSs (65), abnormal beating rate, contractility and calcium-handling properties in cardiomyocytes (169), and susceptibility to methotrexate-induced toxicity in hepatocytes (171). Notably, metabolomics data indicated an increase in nicotinamide nucleotide adenylyltransferase 3 (NMNAT3), a key enzyme in the biosynthesis of NAD+ from ATP, leading to a higher proliferation rate of RA-FLSs-derived iPSCs (65). Inhibition of NMNAT3 by tannic acid led to a reduction in cell proliferation, suggesting that tannic acid might be a potential effective treatment for RA patients (**Table 2**) (65).Togher with data from T1D/SLE-iPSCs-derived cardiomyocytes, we may conclude that diseased cardiomyocytes show more or less abnormal phenotypes although these cells can express normal cardiomyocyte marker, suggesting cardiac complications are a key preventive disease for patients of autoimmune diseases.

In the immunotherapy based on iPSCs-derived Tregs, several studies demonstrated that iPSCs-derived Tregs could infiltrate into the joints and inhibit the arthritis development in the RA animal models (**Table 5**) (22, 148, 149, 172). Besides the transfection of FoxP3 in the section 4.2.6, Bcl-xL, a key anti-apoptotic gene, was also transduced into iPSCs to enhance the differentiation and survival of Tregs in the host (22, 172). The results indicated that the mice received FoxP3+Bcl-xL-transduced Tregs showed a lower arthritis score than FoxP3 alone group (22). In addition, the transfection of TCR was not required for the anti-inflammatory role of iPSCs-derived Tregs, but antigen-specific iPSCs-derived Tregs were more effective to relieve arthritis in the corresponding antigen induced RA model (148). In a recent *in vitro* study, Klimak et al. built an iPSCs-derived macrophage line which was able to express soluble TNF receptor 1 (sTNFR1) through the promoter monocyte chemo-attractant protein-1 (gene name Ccl2, a crucial driver of the recruitment and proliferation of immune cells)-sTNFR1 gene circuit (173). sTNFR1 can bind circulating TNF-α and thereby suppress the inflammatory pathway. The data showed that these macrophages could inhibit the inflammatory response stimulated by TNF-α, LPS and IFN-γ in 24-72 hours (173). And further *in vivo* study should be carried out to evaluate safety and efficacy in the RA animal model because there has the complex microenvironment in the inflamed joints and macrophages also actively participate in the inflammatory process. In summary, iPSCs-derived Tregs is worthy of further study to treat RA and other autoimmune diseases, and some challenges, such as tumorigenicity risk, immune rejection, and large-scale production of Tregs, must be addressed.

**Table 5:** The therapeutic effects of cell therapy based on iPSCs in the treatment of RA.

Report	Cell used	Transplantation routes	Recipient animal	Key findings
Haque et al. (22)	miPSCs-Tregs	Tail vein injection	C57BL/6 or DBA/1 mice	FoxP3 transfected iPSCs could differentiate into Tregs with natural immunoregulatory function. Additional Bcl-xL-transduced iPSCs-derived Tregs might assist with FoxP3 to improve symptoms of arthritis in the RA animal model.
Lei et al. (172)	miPSCs-Tregs	Tail vein injection	C57BL/6 or DBA/1 mice	
Haque et al. (148)	miPSCs-Tregs	Tail vein injection	C57BL/6 mice	Besides FoxP3, additional antigen-specific TCR transduced iPSCs-derived Tregs might be more efficient in the corresponding antigen induced RA model.
Haque et al. (149)	miPSCs-Tregs	Tail vein injection	C57BL/6 mice	

### 5.3 SS

SS is a chronic inflammatory autoimmune disease affecting the lacrimal and salivary glands, leading to symptoms such as dry eyes, itchy eyes, dry mouth, and thirst due to decreased secretion from these glands (174). In a recent study, dendritic cells were generated from iPSCs derived from CD4+ T cells of an SS patient. Functional analysis revealed these cells had similar abilities of phagocytosis, antigen presentation, and stimulation of naive T cells with dendritic cells from mature monocytes of the same patient (175). But results obtained by this study is not very reliable because of too small a sample and no healthy donors.

iPSCs are also used for SS treatment through EVs from iPSCs-derived MSCs (iEVs) (**Table 6**) (176–178). Compared to the EVs from bone marrow-derived MSCs, iEVs have the similar effect in the remission of SS progression and lymphocytes recruitment (176). Similar to paracrine secreted pathway observed in the treatment of IBD by MSCs (91), acting mechanisms of iEVs did not enter salivary glands or cervical lymph nodes, but release active substances to induce more anti-inflammatory M2 polarization of macrophages and inhibit Th17 differentiation in the spleen (178). Notably, the investigators found that young cells-derived iEVs (EVs from a population doubling 15 MSCs) appeared to yield better therapeutic outcomes than those from aging cells (EVs from a population doubling 45 MSCs) (178), indicating that the immune-regulating capacity was relevant with cellular senescence in MSCs (180). Moreover, the hypofunction of EVs is a key hallmark of MSCs aging (180). Some scientists suggested that anti-MSCs aging may be a very useful tool against inflammatory and aging-related diseases by gene modifications and signaling pathways regulation (180, 181). The data of proteomics and microRNA sequencing demonstrated that young cells were rich in the immune regulators TGF-β and miR-125b while aging cells contained more miR-125b, which might emerge as a promising target to intervene the senescence and enhance the therapeutic results in MSCs because inhibiting miR-125b was found to restore the anti-inflammatory capabilities of aging iEVs (178). Even though EVs were derived from the undifferentiated iPSCs, EVs with higher concentrations of HGF, TGF-β1 and let-7 family members could alleviate the SS symptoms by the regulation of MAPK and Smad2/3 pathway in the mice model (**Table 6**) (179). However, three iPSCs lines were prepared in this study (the authors did not describe the different preparation methods in the detail), but only one cell line with abundant HGF and TGF-β1 showed the sufficient therapeutic effect, suggesting that EVs are heterogeneous and the quality depends on some factors, such as donor and culture medium (179). Compared to cell therapy based on iPSCs, as one of cell-free strategies, EVs therapy is a fast-emerging approach with the lower risk of tumorigenicity, immunogenicity and undesired differentiation (182). The people also must pay more attention on the limitations of EVs such as heterogeneity and off-target effects (183).

**Table 6:** The therapeutic effects of cell-free therapy based on iPSCs in the treatment of SS.

Report	EVs used	Transplantation routes	Recipient animal	Key findings
Hai et al. (176)	HiPSCs-MSCs-EVs	Tail vein injection	NOD/ShiLtJ	Both EVs from bone marrow-derived MSCs and iPSCs-derived MSCs had the comparable effect on suppressing the production of inflammatory cytokines and the activation of Th17 cells *in vitro*. Like iPSCs-derived MSCs, iEVs could decrease lymphocyte infiltration in salivary glands and serum autoantibody levels in the SS mice model.
Kim et al. (177)	HiPSCs-MSCs-EVs	Tail vein injection	NOD.B10.H2b mice	The EVs from young but not aging HiPSCs-MSCs could reduce sialadenitis onset through decreasing the secretion of Th1 and Th17 cytokines and increasing the secretion of TGF-β1. Further research identified TGF-β1 and miR-21 as positive anti-inflammatory regulators and miR-125b as a negative regulator, which was proved by the fact that the therapeutic effects were restored by inhibiting miR-125b in aging MSCs.
Zhao et al. (178)	HiPSCs-MSCs-EVs	Tail vein injection	NOD.B10.H2b mice	
Ogata et al. (179)	HiPSCs-EVs	Tail vein injection	NOD mice	The HiPSCs-EVs with rich HGF and TGF-β1 could attenuate SS symptoms by let-7 family-mediated the decreased TLR-4, NF-κB, and phosphorylation-MAPK expression and the up-regulated Smad2/3 signaling pathway.

### 5.4 AS

AS is a form of arthritis that affects the sacroiliac joint and spine, resulting in spinal deformities and a rigid spine. Some studies reported that somatic cells from AS patients were reprogrammed into iPSCs (184–186). Compared to iPSCs and blood cells, AS-iPSCs-derived-MSCs expressed higher levels of several AS susceptibility genes related to antigen presentation, lymphocyte development, immune activation and signaling, ubiquitin-mediated protein regulation, aminopeptidase activity, and so on (184). This indicates that MSCs is closely related to the progression of AS disease. In fact, internal environment of AS patients can result in the dysfunction of MSCs. For example, the morbid serum from AS patients could aggravate cellular senescence leading to function absence in MSCs by oxidative stress-mediated mitochondrial dysfunction (187). Excessive TNF-α levels could induce the abnormal directional migration of MSCs (188). In a mouse periodontitis model (189), loss of MSCs function particularly in differentiation and immunoregulation could exacerbate inflammatory disorders. A small-scale clinical study of 5 patients proved that umbilical cord-derived MSCs had certain curative effect without serious safety issues (190). Hence, supplement of working MSCs could be a promising treatment strategy. The negative results in IBD and SS animal models treated by iPSCs-derived MSCs suggest that a quality standard of ‘working MSCs’ need to be set up in the future.

### 5.5 PsO

PsO is a skin disease characterized by rough red areas where the skin sheds in small pieces, resulting from aberrant proliferation and abnormal differentiation of keratinocytes, and infiltration of multiple inflammatory cells, mainly mediated by the TNF-α and IL-23/IL-17A axes (191, 192). Ali et al. successfully differentiated iPSCs derived from PsO and insulin-resistant patients into mature keratinocytes over 30 days (193). The investigators observed that PsO-iPSCs-derived keratinocytes exhibited dysregulated transcripts associated with psoriasis, keratinocyte differentiation, as well as insulin resistance, providing further evidence of keratinocyte abnormalities driving psoriasis pathology (193). Interestingly, PsO-keratinocytes demonstrated significant insulin resistance with no change in glucose uptake in response to insulin treatment, offering new insights into a potential link between altered gene transcription in psoriatic skin and comorbid conditions (193). As mentioned above, patient-specific iPSCs and iPSCs derivatives dismayed some diseased phenotype through different analytical methods. We can make a bold assumption whether scientists can establish a forward diagnostic system before the onset of symptoms by determining the phenotypes of iPSCs and iPSCs derivatives with or without microenvironment stimuli (such as proinflammatory cytokines and oxidative stress) for high-risk people.

## 6 Discussion

In this article, iPSCs technology offers the potential for novel insights into disease mechanisms, phenotypic determination, new treatment targets, drug discovery, and cell therapy. In these studies, patient or healthy individual-derived iPSCs differentiated into autoimmune diseases-related NPCs, glial cells, pancreatic β cells, intestinal organoids and immune cells. Compared to healthy controls, some cells showed the diseased phenotypes such as senescence, decreased expression of specific markers, aberrant proliferation, abnormal metabolism and transcriptome and susceptibility to inflammatory cytokines, while others such as OPCs and β cells maintained the appropriate structure and function *in vitro* and *in vivo (* 19, 38, 41, 102, 107, 108). In the mechanisms studies based on iPSCs, it is demonstrated that the pro-inflammatory cytokines such as TNF-α, IL-1β, and IFN-γ are the cores for driving disease progression in autoimmune diseases. Furthermore, the deficiency of anti-inflammatory cytokines cannot suppress immunity and aggravate clinical symptoms due to the uncontrolled and elevated release of pro-inflammatory cytokines in the *in vitro* model of iPSCs derivatives (**Figure 2**). This review summarizes the therapeutic effect of iPSCs-based cell therapy and cell-free therapy in autoimmune diseases and these iPSCs derivatives, even from a diseased individual in some studies, can relieve clinical symptoms through cell replacement and immunosuppression in animal models (**Tables 1**, **3**–**6**). The challenges of iPSCs-based cell therapy are solved gradually by optimization of reprogramming method, new biomaterials, and gene-editing technology (**Figure 3**). To reduce reliance on *in vivo* animal models for basic research and therapeutic screening, disease-specific iPSCs and subsequent disease-harboring tissue cells provide a ‘disease in a dish’ model, which preserves endogenous cellular machinery and mutation causing the disease without genetic defects by artificial induction. In drug discovery, iPSC-based studies have identified novel targets such as RNLS and NMNAT3, and some drugs and compounds have shown therapeutic effects (**Table 2**).

Overall, current publications have several shortcomings. Firstly, there is not a consistent conclusion on whether diseased phenotypes can be accurately recapitulated through derived disease-relevant cell types. One limitation is the often-small sample sizes in most studies due to challenges in obtaining clinical samples (194). Consequently, some studies fail to detect meaningful diversity when comparing healthy iPSCs/iPSCs derivatives with diseased iPSCs/iPSCs derivatives, as they often involve a single iPSCs line from one patient versus one control. The small sample size greatly reduces the reliability of the results. Although some studies established a link between disease phenotype and monogenic mutation (43, 79, 163), our another consideration is that iPSCs lose many epigenetic modifications associated with aging, lifestyle, and environmental factors during reprogramming (16, 195, 196), which might be unfavorable for research on autoimmune diseases and explain why patients-iPSCs-derived somatic cells do not consistently exhibit significant differences from controls. An alternative approach will be direct conversion of one somatic cell type to another without intermediate pluripotent stage through inducing transcription factors and miRNAs (16), which may be a useful technique for phenotypic identification and drug testing. Moreover, while transcriptomics, proteomics, and metabolomics studies generate vast amounts of data highlighting phenotypic differences between diseased and control cells, it does not guarantee that the pathways, kinases, or receptors associated with these differences are effective biomarkers or therapeutic targets. Differential proteins need the large samples to determine if it meets the criteria for a biomarker in accuracy and sensitivity which will spend a lot of manpower and wealth. It is necessary to combine patient-derived iPSCs derivatives with post-mortem samples and animal models to find more valuable autoimmune disease-relevant phenotypes and mutations. For cell therapy based on iPSCs, the infinite proliferative potential of iPSCs grants an unlimited supply of iPSCs derivatives. But we still face three important challenges. Firstly, cell replacement therapy may not be suitable for treatment of autoimmune diseases. Autoimmune diseases are systemic and immune cells play an important role in disease progression. It is possible that transplanted cells will be attacked by autoimmune system again. Also damaged organs are extensive, and it is hard to confirm the best transplantation site and cell type like MS. iPSCs-based immunotherapy, Tregs and regulatory dendritic cells, tends to have the durable treatments with a long-term tolerance. Therefore, we believe that iPSCs-based immunotherapy, may be a better treatment strategy than cell replacement. In addition, some further considerations such as rigorous GMP quality system and high cost should be resolved. Secondly, almost all autoimmune diseases are polygenic disorders (194). However, the current gene-editing technology lack effective strategies for editing multiple genome sites at the same time, which limits their application in complex genomics and polygenic diseases (197). Hence, this may of modifying disease-susceptibility genes in iPSCs by gene-editing is still impractical. Thirdly, the heterogeneity of iPSCs derivatives remains poorly understood, although we know some factors that may reduce the therapeutic effect, such as donors and aging (46, 178). For example, some studies showed that iPSCs-derived MSCs demonstrated a weaker therapeutic effect than MSCs from other sources (92). This could limit iPSCs derivatives clinical applications and experimental reproducibility and consistency. Emerging technologies, such as Single-cell RNA sequencing, hold promise in comprehensively understanding cellular heterogeneity and identifying crucial subpopulations in cell therapy, thereby enhancing iPSCs-based therapeutic efficacy.

And surely our goal is to develop novel and therapeutic approaches in autoimmune diseases through iPSCs technology. Compared to neurodegenerative diseases, autoimmune diseases-relevant basic and clinical research based on iPSCs are still in beginning stage, there is still a long way to go from bench to bedside. However, with the development of technology, it is believable that iPSCs may offer the way forward to mitigating autoimmune diseases in humans.

## Author contributions

RR: Writing – original draft. JJ: Writing – review & editing. XL: Writing – review & editing. GZ: Supervision, Writing – review & editing.
